# Temperature dependences of surface tension, density and viscosity study of Sn-Ag-Cu with Bi additions using theoretical models

**DOI:** 10.1038/s41598-019-50698-9

**Published:** 2019-10-02

**Authors:** Rachida M’chaar, Abdelaziz Sabbar, Mouloud El Moudane

**Affiliations:** 1 Center of Water, Natural Resources, Environment and Sustainable Development, Laboratory of Spectroscopy Molecular Modeling Nanomaterial Materials Water and Environment University of Mohammed V-Rabat, Faculty of Sciences, Ibn Battouta Av, PB 1014 Rabat, Morocco; 2 Departement of chemistry, laboratory of materials, Nanotechnology and Environment, University of Mohammed V, Faculty of Sciences, Av. Ibn Battuta, PB 1014 Rabat, Morocco

**Keywords:** Chemistry, Environmental chemistry

## Abstract

In this work, the kohler, Muggianu,Toop and Hillert geometric models were used to calculate the surface tension, molar volume and density of the liquid Sn-Ag-Cu-Bi quaternary alloys along three selected sections *x*_Sn_:*x*_Ag_:*x*_Cu_ = 1:1:1, 1:1:2 and 1:2:1 in the temperature range of 923 K–1423 K. The choice of this temperature range was made on the basis of the calculation results of the liquidus line of the alloys belonging to the three sections. The same properties have been estimated for five selected Sn2.7Ag0.86Cu3.86Bi, Sn3.13Ag0.48Cu4.02Bi, Sn2.95Ag0.53Cu6.81Bi, Sn2.68Ag1.01Cu6.62Bi and Sn3.24Ag0.75Cu1.76Bi quaternary alloys between 623 K and 1123 K for comparison with the available experimental data. Moreover, the surface tension and density of these five alloys have also been calculated on the basis of Guggenheim and theoretical equation, respectively. In addition, the Seetharaman-sichen and Kaptay equations were extended to estimate the viscosity of SAC + Bi alloys. We also discussed the influence of Bismuth addition in liquid Sn-Ag-Cu-Bi. Estimated values show that Bi increases molar volume and density but decreases the surface tension and viscosity. On the other hand, the surface tensions diminish with the temperature for the all studied models, with the exception of some concentration of Bismuth; an inverse tendency is observed (dσ/d*T*) > 0. While, the density diminishes with increasing temperature for all alloys (dσ/d*T*) < 0. These models have been shown to be a great alternative for calculating the thermo-physical properties of quaternary systems.

## Introduction

In recent years, researchers have been very interested in identifying alternatives for lead solders because they are harmful to the environment. This trend has been encouraged in Europe by the RoHS and WEEE European Directive. Based on the analysis of several multi-component systems, two sets of alloys are potential substitutes for lead soldering. The first groups are the Sn-Ag-Cu (SAC) alloys with the addition of different metals (Bi, Zn, In and Sb)^1–6^. Previous studies have recommended the Sn-Ag-Cu-Bi quaternary system as a promising candidate for lead-free solders, as the biggest advantage of SnAgCuBi over welds SnAgCu is the lower melting temperature. In SnAgCuBi, bismuth plays an important role in lowering the melting temperature of the alloy. It is possible to reduce the melting temperature of the alloy from 217 °C to 208 °C, by balancing the amount of bismuth as well as the amounts of silver and copper in the alloys. However, the addition of Bi to Sn-Ag-Cu alloys increases the mechanical properties of solders^7^.

Since surface properties play a key role in the development of welding, the development of new welds requires data on thermophysical properties such as surface tension that can be obtained experimentally or by numerical modeling if reliable data exist for subsystems and pure components. In addition, theoretical model is sometimes preferred because of the increasing complexity and costs of experimentation, especially for multi-component systems (quaternary, quinary…). The analysis of the influence of the amount of bismuth in the Sn-Ag-Cu-Bi quaternary welds on various properties was carried out by Hwang^8^ and Takao *et al*.^9^. However, little previous work on thermo-physical and electrical properties has been reported in the literature^10^. Indeed, Moser *et al*.^11^, in 2006, have measured the thermo-physical and mechanical proprieties of Sn-Ag-Cu- eutectic alloy with different additions of Bismuth using different techniques. They showed a linear dependence of surface tension as a function of temperature. At the same time, Ohnuma *et al*.^12^ have studied the surface tension of Sn-Ag-Cu-Bi liquid alloys using Butler equation^13^. Their results have been compared with some experimental values^11^. Later, Gancarz *et al*.^14^ have measured the surface tension, density and viscosity of Sn2.92 Ag 0.4Cu 3.07 Bi liquid alloy as a function of temperature by using different methods. The obtained results showed that the addition of Bi to the SAC increases the density and decreases the surface tension and the viscosity. In addition, they showed that the properties studied by different methods (maximum bubble pressure, dilatometry, capillary flow and discharge crucible) were almost similar.

In the present paper, we presented the theoretical results of the surface tension, molar volume and density of Sn-Ag-Cu-Bi system using different geometric models, Kohler^15^, Muggianu^16^, Toop^17^ and Hillert^18^. The geometric models are used in this work in order to verify their effectiveness since they are considered as the most widespread theoretical models used for metallic alloys, especially for ternary systems. As well as, surface tension and density predicted by the Guggenheim^19^ and theoretical equations^20^, respectively. In addition, we presented the theoretical results of viscosity using the Seetharaman-Sichen^21^ and Kaptay equations^22^ and the predicted results are compared to each other and to the experimental ones^11,14^.

Our calculations were done along three cross-sections *x*_Sn_:*x*_Ag_:*x*_Cu_ = 1:1:1, 1:1:2 and 1:2:1 between 923 K and 1423 K and Sn2.7Ag0.86Cu3.86Bi, Sn3.13Ag0.48Cu4.02Bi, Sn2.95Ag0.53Cu6.81Bi, Sn2.68Ag1.01Cu6.62Bi and Sn3.24 Ag0.75 Cu1.76 Bi quaternary alloys in the temperature range 623 K–1123 K.

## Method of Calculation

Numerous studies have been carried out for the creation of adequate databases of thermo-physical properties (surface tension, density and viscosity) of Sn, Ag, Cu and Bi under the framework of developing lead-free solders. In this work, we used the pure constituents of Sn, Ag, Cu and Bi inside the temperature range (523 K–1473 K) of our prediction. Moreover, among these numerous studies, we have chosen the values for pure elements that led to values of quaternary system Sn-Ag-Cu-Bi closest to the experimental ones.

### Modeling surface tension of liquid Sn-Ag-Cu-Bi system from geometric models

The information of the properties of binary alloys is indispensable for the prediction of thermo-physical properties of quaternary alloys for the purpose of comparing with experimental data. The models proposed by various researchers are exposed in the following sections. The models of Kohler^15^ and Muggianu *et al*.^16^ were used in this work as simple models based on binary data, while the Toop^17^ and Hillert^18^ models are asymmetric.

#### Kohler model

The Kohler and Muggianu models have been used to estimate the surface tension of Sn-Ag-Cu-Bi quaternary alloys. Consequently, the properties of any quaternary system can be calculated from the knowledge of the corresponding properties of the boundary binaries.

Indeed, for the two symmetric models, the surface tension for a four-component system has been expressed by the following equations:

1 $$\begin{array}{ccc}{\sigma }_{1-2-3-4}^{E} & = & {({x}_{1}+{x}_{2})}^{2}.{\sigma }_{12}^{E}(\frac{{x}_{1}}{{x}_{1}+{x}_{2}},\frac{{x}_{2}}{{x}_{1}+{x}_{2}})+{({x}_{1}+{x}_{3})}^{2}.{\sigma }_{13}^{E}(\frac{{x}_{1}}{{x}_{1}+{x}_{3}},\frac{{x}_{3}}{{x}_{1}+{x}_{3}})\\  &  & +\,{({x}_{1}+{x}_{4})}^{2}.{\sigma }_{14}^{E}(\frac{{x}_{1}}{{x}_{1}+{x}_{4}},\frac{{x}_{4}}{{x}_{1}+{x}_{4}})+{({x}_{2}+{x}_{3})}^{2}.{\sigma }_{23}^{E}(\frac{{x}_{2}}{{x}_{2}+{x}_{3}},\frac{{x}_{3}}{{x}_{2}+{x}_{3}})\\  &  & +\,{({x}_{2}+{x}_{4})}^{2}.{\sigma }_{24}^{E}(\frac{{x}_{2}}{{x}_{2}+{x}_{4}},\frac{{x}_{4}}{{x}_{2}+{x}_{4}})+{({x}_{3}+{x}_{4})}^{2}.{\sigma }_{34}^{E}(\frac{{x}_{3}}{{x}_{3}+{x}_{4}},\frac{{x}_{4}}{{x}_{3}+{x}_{4}})\end{array}$$

*Muggianu model.*

2 $$\begin{array}{c}{\sigma }_{1-2-3-4}^{E}=\frac{4{x}_{1}{x}_{2}}{(1+{x}_{1}-{x}_{2})(1+{x}_{2}-{x}_{1})}.{\sigma }_{12}^{E}(\frac{1+{x}_{1}-{x}_{2}}{2},\frac{1+{x}_{2}-{x}_{1}}{2})\\ +\,\frac{4{x}_{1}{x}_{3}}{(1+{x}_{1}-{x}_{3})(1+{x}_{3}-{x}_{1})}.{\sigma }_{13}^{E}(\frac{1+{x}_{1}-{x}_{3}}{2},\frac{1+{x}_{3}-{x}_{1}}{2})\\ +\,\frac{4{x}_{1}{x}_{4}}{(1+{x}_{1}-{x}_{4})(1+{x}_{4}-{x}_{1})}.{\sigma }_{14}^{E}(\frac{1+{x}_{1}-{x}_{4}}{2},\frac{1+{x}_{4}-{x}_{1}}{2})\\ +\,\frac{4{x}_{2}{x}_{3}}{(1+{x}_{2}-{x}_{3})(1+{x}_{3}-{x}_{2})}.{\sigma }_{23}^{E}(\frac{1+{x}_{2}-{x}_{3}}{2},\frac{1+{x}_{3}-{x}_{2}}{2})\\ +\,\frac{4{x}_{2}{x}_{4}}{(1+{x}_{2}-{x}_{4})(1+{x}_{4}-{x}_{2})}.{\sigma }_{24}^{E}(\frac{1+{x}_{2}-{x}_{4}}{2},\frac{1+{x}_{4}-{x}_{2}}{2})\\ +\,\frac{4{x}_{3}{x}_{4}}{(1+{x}_{3}-{x}_{4})(1+{x}_{4}-{x}_{3})}.{\sigma }_{34}^{E}(\frac{1+{x}_{3}-{x}_{4}}{2},\frac{1+{x}_{4}-{x}_{3}}{2})\end{array}$$

#### Toop model

On the other hand, the asymmetric models of Toop and Hillert are respectively expressed by the two equations:

3 $$\begin{array}{rcl}{\sigma }_{1-2-3-4}^{E} & = & \frac{{x}_{2}}{1-{x}_{1}}.{\sigma }_{12}^{E}({x}_{1},1-{x}_{1})+\frac{{x}_{3}}{1-{x}_{1}}.{\sigma }_{13}^{E}({x}_{1},1-{x}_{1})\\  &  & +\,\frac{{x}_{4}}{1-{x}_{1}}.{\sigma }_{14}^{E}({x}_{1},1-{x}_{1})+{({x}_{2}+{x}_{3})}^{2}\cdot {\sigma }_{23}^{E}(\frac{{x}_{2}}{{x}_{2}+{x}_{3}},\frac{{x}_{3}}{{x}_{2}+{x}_{3}})\\  &  & +\,{({x}_{2}+{x}_{4})}^{2}{\sigma }_{24}^{E}(\frac{{x}_{2}}{{x}_{2}+{x}_{4}},\frac{{x}_{4}}{{x}_{2}+{x}_{4}})+{({x}_{3}+{x}_{4})}^{2}{\sigma }_{34}^{E}(\frac{{x}_{3}}{{x}_{3}+{x}_{4}},\frac{{x}_{4}}{{x}_{3}+{x}_{4}})\end{array}$$

*Hillert model*.

4 $$\begin{array}{rcl}{\sigma }_{1-2-3-4}^{E} & = & \frac{{x}_{2}}{1-{x}_{1}}.{\sigma }_{12}^{E}({x}_{1},1-{x}_{1})+\frac{{x}_{3}}{1-{x}_{1}}.{\sigma }_{13}^{E}({x}_{1},1-{x}_{1})\\  &  & +\,\frac{{x}_{4}}{1-{x}_{1}}.{\sigma }_{14}^{E}({x}_{1},1-{x}_{1})+\frac{{x}_{2}.{x}_{3}}{{\nu }_{23}.{\nu }_{32}}.{\sigma }_{23}^{E}({\nu }_{23},{\nu }_{32})\\  &  & +\,\frac{{x}_{2}.{x}_{4}}{{\nu }_{24}.{\nu }_{42}}.{\sigma }_{24}^{E}({\nu }_{24},{\nu }_{42})+\frac{{x}_{3}.{x}_{4}}{{\nu }_{34}.{\nu }_{43}}.{\sigma }_{34}^{E}({\nu }_{34},{\nu }_{43})\end{array}$$

with $${\nu }_{23}=(\frac{1+{x}_{2}-{x}_{3}}{2})$$ and $${\nu }_{32}=(\frac{1+{x}_{3}-{x}_{2}}{2})$$

In all equations ${\sigma }_{234}^{E}$ and ${\sigma }_{1-2-3-4}^{E}$ correspond to the excess surface tension for ternary and quaternary system, while *x*_1_, *x*_2_, *x*_*3*_ and *x*_*4*_ represent the mole fractions of components in investigated system.

#### The excess surface tension of six binary systems

The excess surface tension σ^*E*^ for binary system is a composition dependency of surface tension of mixture that can be defined as follows:

5 $${\sigma }^{E}=\sigma -{\sigma }^{i}$$

with *σ* and *σ*^*i*^ represent the surface tension of binary alloys and the surface tension of the ideal alloys, respectively.

*σ*^*i*^ can be defined as following:

6 $${\sigma }^{i}={X}_{1}{\sigma }_{1}+{X}_{2}{\sigma }_{2}$$

with *X*_i_ and *σ*_*i*_ represent the molar fraction of a constituent i and the surface tension of the pure constituent i, respectively. The surface tension, as a function of temperature, of the pure constituent i is presented in Table 1.

**Table 1:** Surface tension of the pure constituents.

Constituents	σ_i_ (mN/m)	Reference
Sn	636.5–0.1101 T	^23^
Ag	1164–0.204 T	^25^
Cu	1475.648–0.14224 *T*	^24^
Bi	431–0.08 T	^23^

The excess surface tension values of the six binary alloys are taken from previous work Sn-Ag^23^, Sn-Cu^24^, Sn-Bi^23^, Ag-Cu^25^ and Ag-Bi^23^. The values of Cu-Bi sub-binary alloys are predicted based on Butler’s equation^13^.

### Using of guggenheim equation

Based on the model of regular solutions, the Guggenheim^19^ equation relies on statistical approximations, in which the constituents of the binary are distributed randomly within the quasi-crystalline liquid. Moreover, this equation has been developed for quaternary alloys, which can be written in the form:

7 $$\exp (-\frac{{\sigma }_{{\rm M}}A}{RT})={x}_{1}\,\exp (-\frac{{\sigma }_{1}{A}_{1}}{RT})+{x}_{2}\,\exp (-\frac{{\sigma }_{2}{A}_{2}}{RT})+{x}_{3}\,\exp (-\frac{{\sigma }_{3}{A}_{3}}{RT})+{x}_{4}\,\exp (-\frac{{\sigma }_{4}{A}_{4}}{RT})$$

where *σ*_*M*_ is the surface tension of the quaternary system, σ_1_, σ_2_, σ_3_ and σ_4_ are surface tensions of the individual components of the alloy, and A is the molar surface area which is defined by:

8 $$A=f{N}_{A}^{1/3}{(\frac{M}{\rho })}^{2/3}$$

$$\rho =\sum {x}_{i}{\rho }_{i}$$ and $$M=\sum {x}_{i}{M}_{i}$$

*ρ* and *ρ*_i_ represent the densities of quaternary alloys and pure constituent i, respectively. *M* and *M*_*i*_ are atomic weights of the quaternary alloys and pure constituent i, respectively (Table 2). *N*_A_ is the Avogadro number and *f* is the atomic arrangement factor for the liquid surface.

**Table 2:** Data on density and atomic mass of the pure constituents

Constituents	*ρ*_*i*_*(g/cm^*3*)*	*M*_*i*_ *(g/mol)*	*Reference*
*Sn*	7.118–0.00051 *T*	118.6900	^37^
*Ag*	10.18–0.00071 *T*	107.8680	^25^
*Cu*	9.405–0.001 *T*	63.546	^23^
*Bi*	10.73–0.0012 *T*	208.9804	^38^

### The excess molar volume of six binary systems

In this research, we used Kohler^15^, Muggianu^16^, Toop^17^ and Hillert^18^ to calculate molar volume of Sn-Ag-Cu-Bi alloys. The excess molar volume, *V*^*E*^ for binary system is a composition dependency of molar volume of mixture that can be defined as follows:

9 $${V}^{E}=V-{V}^{i}$$

*V* and *V*^*i*^ represent the molar volume of binary alloys and the molar volume of the ideal alloys, respectively.

*V*^*i*^ can be defined as follow:

10 $${V}^{i}={X}_{1}{V}_{1}+{X}_{2}{V}_{2}$$

*X*_i_ and *V*_*i*_ represent the molar fraction of a constituent i and the molar volume of the pure constituent i, respectively (Table 3).

**Table 3:** Molar volume of the pure constituents.

Constituents	*V*_*i*_ (m^3^/mol)	*Reference*
Sn	17.0.10^−6^(1.0 + 0.000087(*T*-504.99))	^39^
Ag	11.6.10^−6^.(1.0 + 0.000098(*T*-1234))	^40^
Cu	7.94. 10^−6^.(1.0 + 0.00001(*T*-1357.77))	^41^
Bi	20.80.10^−6^ (1.0 + 0.000117(*T*-544.1))	^25^

The excess molar volume values of the sub-binary systems are taken from previous work Sn-Ag^23^, Sn-Cu^24^, Sn-Bi^23^, Ag-Cu^25^ and Ag-Bi^23^.

The molar volume of Cu-Bi binary system was estimated using:

11 $${V}_{M}=\frac{\sum {x}_{i}{M}_{i}}{{\rm{\rho }}}$$

ρ represents the density of binary Cu-Bi alloys. *x*_*i*_ and *M*_*i*_ are the molar fraction and molar mass of constituent i, respectively.

### Calculation of molar volume of liquid phase in the Sn-Ag-Cu-Bi system

The same equations previously used for the calculation of the excess surface tension *σ*^*E*^ will be used for the calculation of the excess molar volume *V*^*E*^.

### Density calculation of liquid the Sn-Ag-Cu-Bi system

The relationship between density and molar volume is an example is expressed as:

12 $${\rm{\rho }}=\frac{\sum {x}_{i}{M}_{i}}{{V}_{M}}$$

where ρ is the density of mixture, while *x*_*i*_ and *M*_*i*_ are the molar fraction and molar mass of component *i. V*_*M*_ is a molar volume of a mixture, calculated using Kohler^15^, Muggianu^16^, Toop^17^ and Hillert^18^ models.

The mathematical prediction of density is based on semi-empirical equations as well as on theoretical equations^20^, ever since the atomic volumes of most liquid binary alloys is in fact a linear equation of composition, the calculation of alloy density can be approximated by an addition. A similar procedure can be used for higher order alloys, as indicated by:

13 $${\rm{\rho }}={x}_{1}{{\rm{\rho }}}_{1}+{x}_{2}{{\rm{\rho }}}_{2}+{x}_{3}{{\rm{\rho }}}_{3}+{x}_{4}{{\rm{\rho }}}_{4}$$

where *x*_1_, *x*_2_, *x*_3_, *x*_4_ are the atomic fractions of the constituents of the alloys, ρ_1_, ρ_2_ρ_3_ and ρ_4_ are the densities of the pure components (Table 2).

### Viscosity in the liquid Sn-Ag-Cu-Sn quaternary alloys

Viscosity is one of the key properties of alloys that influence the performance of pyrometallurgical processes in several ways. As such, the researcher has made considerable efforts in numerous experimental studies to quantify the dependence on viscosity composition and temperature of many simple and complex alloy systems. However, because of the problems and difficulties inherent in high temperature measurements, the available experimental results only cover a limited range of compositions and temperatures and do not fully meet the needs of the industry. In addition, the accuracy or reliability of some published data has been found to be unsatisfactory. The discrepancy between some of the data is significantly large^26^.

As a result, in recent decades, various models have been allocated to calculate the viscosity of metal alloys. These models are based on one of the theoretical equations used to estimate the viscosity of single liquids, such as Eyring’s equation^27^, the Bockris Bockris’ equation^28^, Weymann’s equation^29^ and Frenkel’s equation^30^. There have also been some studies to correlate viscosity with self-defined parameters to find some consistency^26^. All modeling and correlation studies were useful and provided a reasonably good description of viscosity over a range of temperatures and compositions.

#### Seetharaman-Sichen equation

The Seetharaman-Du Sichen equation^21^ is a mathematical equation for estimation the viscosity used and developed for quaternary Sn-Ag-Cu-Bi alloys:

14 $$\begin{array}{c}\eta =\frac{h{N}_{0}\rho }{M}\exp (\frac{\Delta {G}^{\ast }}{RT})\\ \rho =\sum {x}_{i}{\rho }_{i}\,{\rm{and}}\,M=\sum {x}_{i}{M}_{i}\end{array}$$

where *ρ* and *ρ*_i_ are the density of liquid alloys and pure component i, respectively. *M* and *M*_*i*_ are atomic weights of the liquid alloy and pure component i, respectively, *h* is Planck’s constant. *N*_*Av*_ is Avogadro’s number. ΔG* represents the Gibbs activation energy for viscosity. We can express this energy in the form:

15 $$\Delta {G}^{\ast }=\sum {x}_{i}\Delta {G}_{i}^{\ast }+3RT\sum {x}_{i}{x}_{j}+RT\sum {x}_{i}\mathrm{ln}\,{x}_{i}+\Delta {G}_{1-2-3-4}^{E}(T,{x}_{B})$$

where $\Delta {G}_{i}^{\ast }\,$is the Gibbs activation energy of the viscous flow in pure constituent *i*

16 $$\Delta {G}_{i}^{\ast }=RTLn(\frac{{\eta }_{i}{M}_{i}}{h{N}_{0}{\rho }_{i}})$$

*ŋ*_i_ is the viscosity of the pure constituent i (Pa.s) (Table 4) and R = 8.314 J.mol^−1^K^−1^ and $\Delta {G}_{1-2-3-4}^{E}(T,{x}_{B})$ is the excess energy of alloy in the liquid phase for quaternary alloys.

**Table 4:** Data on viscosity of the pure constituents.

Constituents	*ŋ*_*i*_ *(mPa.s)*	*Reference*
*Sn*	$0.4475\mathrm{Exp}(\frac{61914.213}{RT})$	^42^
*Ag*	$0.5976291\mathrm{Exp}(\frac{19136.85}{RT})$	^37^
*Cu*	$1.769\mathrm{Exp}(\frac{10833.716}{RT})$	^43^
*Bi*	$0.445\mathrm{Exp}(\frac{6450}{{\rm{RT}}})$	^38^

This excess Gibbs energy was calculated using the Kohler geometric^15^.

17 $$\begin{array}{rcl}\Delta {G}_{1-2-3-4}^{E} & = & {({x}_{1}+{x}_{2})}^{2}.\Delta {G}_{12}^{E}(\frac{{x}_{1}}{{x}_{1}+{x}_{2}},\frac{{x}_{2}}{{x}_{1}+{x}_{2}})+{({x}_{1}+{x}_{3})}^{2}.\Delta {G}_{13}^{E}(\frac{{x}_{1}}{{x}_{1}+{x}_{3}},\frac{{x}_{3}}{{x}_{1}+{x}_{3}})\\  &  & +\,{({x}_{1}+{x}_{4})}^{2}.\Delta {G}_{14}^{E}(\frac{{x}_{1}}{{x}_{1}+{x}_{4}},\frac{{x}_{4}}{{x}_{1}+{x}_{4}})+{({x}_{2}+{x}_{3})}^{2}.\Delta {G}_{23}^{E}(\frac{{x}_{2}}{{x}_{2}+{x}_{3}},\frac{{x}_{3}}{{x}_{2}+{x}_{3}})\\  &  & +\,{({x}_{2}+{x}_{4})}^{2}.\Delta {G}_{24}^{E}(\frac{{x}_{2}}{{x}_{2}+{x}_{4}},\frac{{x}_{4}}{{x}_{2}+{x}_{4}})+{({x}_{3}+{x}_{4})}^{2}.\Delta {G}_{34}^{E}(\frac{{x}_{3}}{{x}_{3}+{x}_{4}},\frac{{x}_{4}}{{x}_{3}+{x}_{4}})\end{array}$$

$\Delta {G}_{12}^{{\rm{E}}}\,,\Delta {G}_{13}^{{\rm{E}}},\Delta {G}_{14}^{{\rm{E}}},\Delta {G}_{23}^{{\rm{E}}},\Delta {G}_{24}^{{\rm{E}}}\,{\rm{and}}\,\Delta {G}_{34}^{{\rm{E}}}$ represent the excess Gibbs energies of the boundary binary systems taken along the quasi-binary sections *X*_*i*_/*X*_*j*_* = x*_*i*_/*x*_*j*_. *x*_*i*_ and *Xi* are respectively the molar fraction of a constituent i in the quaternary and binary system.

The contribution of the six binary systems for the Sn-Ag-Cu-Bi quaternary alloy is described by polynomial Redlich-Kister polynomial^31^:

18 $$\begin{array}{c}\Delta {G}_{12}^{E}={X}_{1}{X}_{2}({L}_{12}^{0}+{L}_{12}^{1}({X}_{1}-{X}_{2})+{L}_{12}^{2}{({X}_{1}-{X}_{2})}^{2})\\ \Delta {G}_{13}^{E}={X}_{1}{X}_{3}({L}_{13}^{0}+{L}_{13}^{1}({X}_{1}-{X}_{3})+{L}_{13}^{2}{({X}_{1}-{X}_{3})}^{2})\\ \Delta {G}_{14}^{E}={X}_{1}{X}_{4}({L}_{14}^{0}+{L}_{14}^{1}({X}_{1}-{X}_{4})+{L}_{14}^{2}{({X}_{1}-{X}_{4})}^{2})\\ \Delta {G}_{23}^{E}={X}_{2}{X}_{3}({L}_{23}^{0}+{L}_{23}^{1}({X}_{2}-{X}_{3})+{L}_{23}^{2}{({X}_{2}-{X}_{3})}^{2})\\ \Delta {G}_{24}^{E}={X}_{2}{X}_{4}({L}_{24}^{0}+{L}_{24}^{1}({X}_{2}-{X}_{4})+{L}_{24}^{2}{({X}_{2}-{X}_{4})}^{2})\\ \Delta {G}_{34}^{E}={X}_{3}{X}_{4}({L}_{34}^{0}+{L}_{34}^{1}({X}_{3}-{X}_{4})+{L}_{34}^{2}{({X}_{3}-{X}_{4})}^{2})\end{array}$$

${{\rm{L}}}_{12}^{0}\,,{{\rm{L}}}_{12}^{1},{{\rm{L}}}_{12}^{2},{{\rm{L}}}_{13,}^{0}{{\rm{L}}}_{13}^{1},{{\rm{L}}}_{13}^{2},{{\rm{L}}}_{14}^{0}{,L}_{14}^{1},{{\rm{L}}}_{14}^{2},{{\rm{L}}}_{23}^{0},{{\rm{L}}}_{23}^{1},{{\rm{L}}}_{23}^{2}\,,,{{\rm{L}}}_{24}^{0},{{\rm{L}}}_{24}^{1},{{\rm{L}}}_{24}^{2}\,{\rm{and}},{{\rm{L}}}_{34}^{0},{{\rm{L}}}_{34}^{1},{{\rm{L}}}_{34}^{2}$ being the interaction parameters of the binary systems that depend on the temperature.

#### Kaptay equation

The Kaptay equation^22^ is a modification of the Seetharaman-Du equation Sichen^21^, takes into account the theoretical relationship between the energy of cohesion alloys and the Gibbs activation energy of the viscous flow^32^. It has for expression:

19 $$\eta =\frac{h{N}_{Av}}{\sum _{i}{x}_{i}{V}_{i}+\Delta {V}^{E}}\exp [\sum _{i}\frac{{x}_{i}\Delta {G}_{i}^{\ast }-\alpha \Delta {H}_{mix}}{RT}]$$

where Δ*V*^*E*^ is the molar volume of excess for liquid quaternary alloys (m^3^/mol) and Δ*H*_*mix*_ represent the integral enthalpy of the mixture, α is a semi-empirical parameter of the model. It is worth (0.155 ± 0.015) and can be neglected when experimental data are not available^32^.

Most thermodynamic data of ternary and multicomponent systems will come from a theoretical calculation rather than direct experiences because of their difficulties especially for metallurgical systems. The geometric model has been applied to estimate the integral enthalpy of mixing. Chou^33^ has presented a general geometric model for calculating the thermodynamic properties of ternary and multi-component systems from binary data^32^.

## Results and Discussion

### Surface tension in the liquid Sn-Ag-Cu-Bi quaternary alloys

The phase equilibria in the Sn-Ag-Bi-Cu quaternary system have been studied theoretically using thermodynamic calculations for three sections *x*_Sn_:*x*_Ag_:*x*_Cu_ = 1:1:1, 1:1:2 and 1:2:1 and 1:2:1. The Gibbs energy values and the interaction parameters of all the phases of this system have been taken from NIST solder database^34^. The calculations were performed using Open Calphad software^35^.

**Figure 1:**
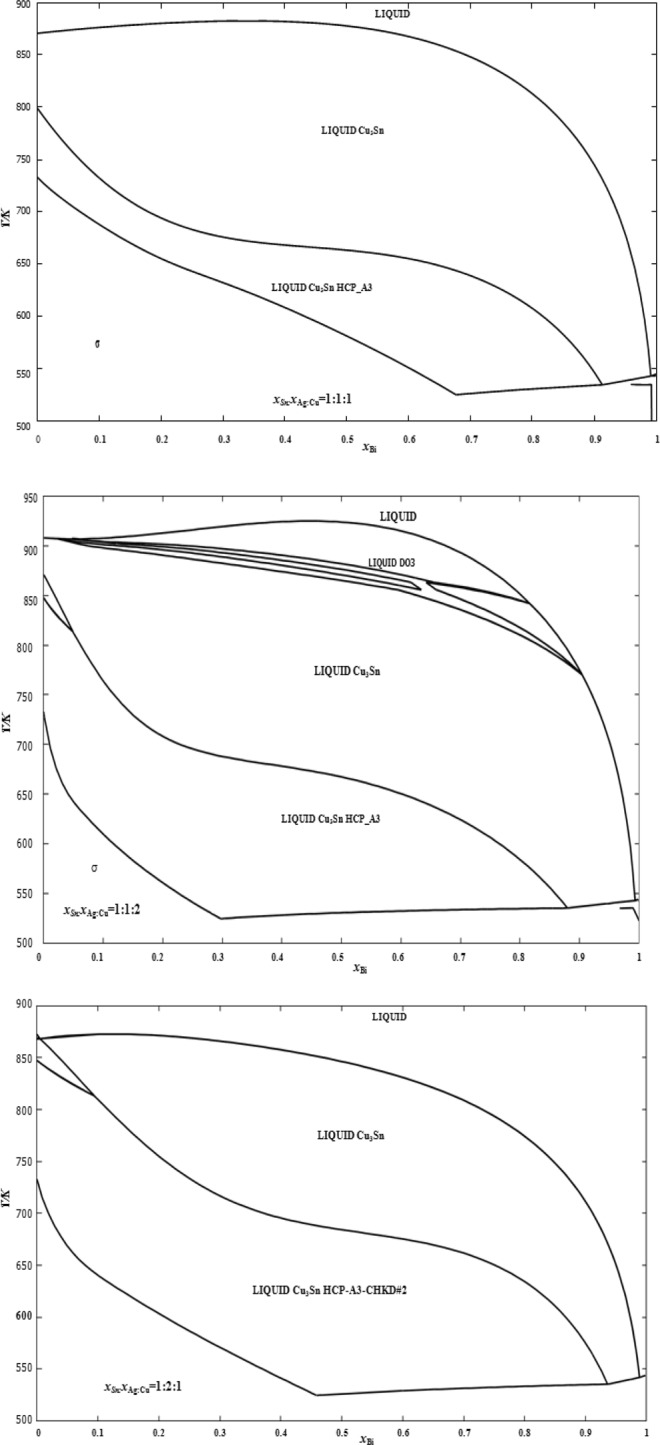
Calculated phase diagrams for the Sn-Ag-Cu-Bi along three selected sections of *x*_Sn_:*x*_Ag_:*x*_Cu_ = 1:1:1, 1:1:2 and 1:2:1.

As shown in Fig. 1, the results show that above 923 K, all the quaternary alloys of the three studied sections are in the liquid phase.

One of the objectives of this article is to show that the geometric models and the Guggenheim equation work well for poly-constituent systems, especially for the quaternaries. Our calculations were done along three ternary sections Sn:Ag:Cu = 1:1:1,1:1:2 and 1:2:1. In addition, (Sn-Ag-Cu)_eutc_ + Bi have been done using different predicting methods such as Kohler^15^, Muggianu^16^, Toop^17^ and Hillert^18^ models and Guggenheim equation^19^ in the temperature range from 923 K to 1423 K.

**Figure 2:**
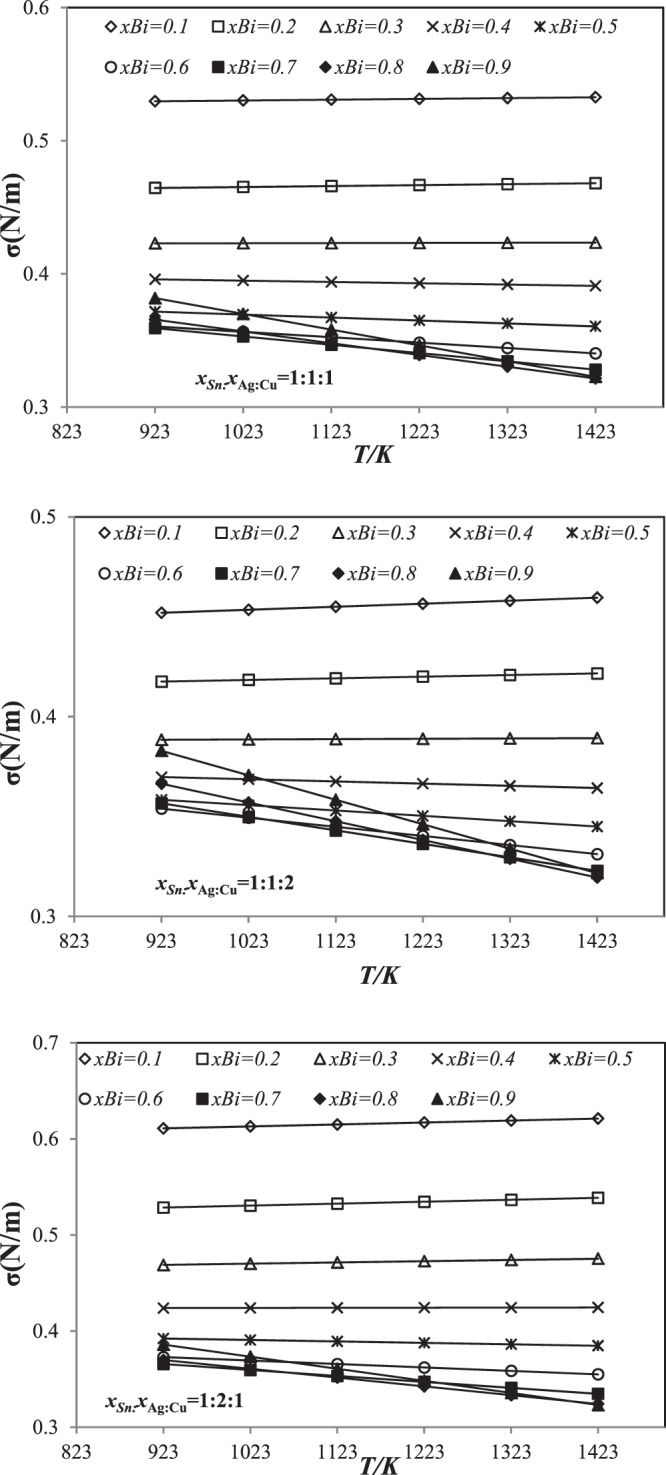
Linear dependence of surface tension for three sections *x*_Sn_:*x*_Ag_:*x*_Cu_ = 1:1:1, 1:1:2 and 1:2:1 using Kohler’s model from 923 *K* to 1423 *K*.

**Figure 3:**
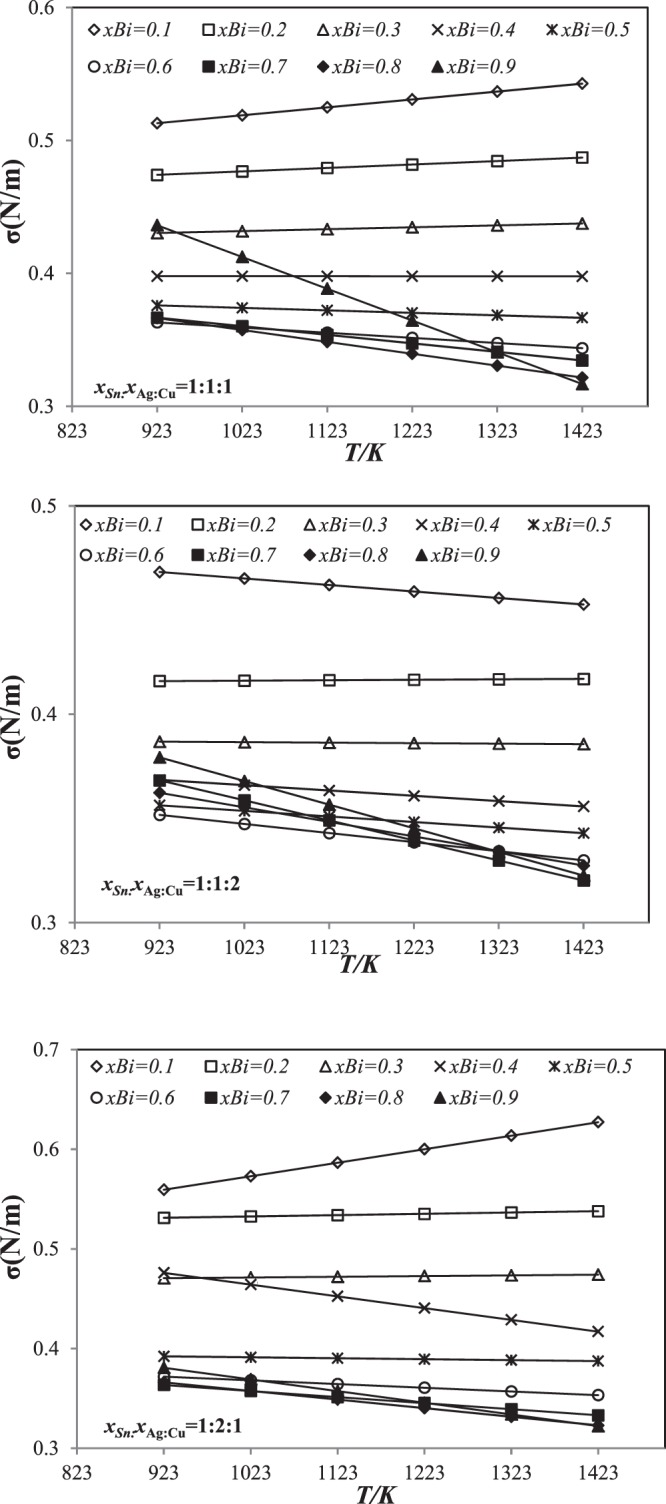
Linear dependence of surface tension for three sections *x*_Sn_:*x*_Ag_:*x*_Cu_ = 1:1:1, 1:1:2 and 1:2:1 using Toop’s model from 923 *K* to 1423 *K*.

The linear equations describing the temperature dependence of the surface tension for Sn-Ag-Cu-Bi quaternary alloys are illustrated in Figs 2 and 3 showing the results obtained according to Kohler and Toop models along three cross sections *x*_Sn_:*x*_Ag_:*x*_Cu_ = 1:1:1, 1:1:2 and 1:2:1 over a wide range of temperatures, between 923 and 1423 K. For most of compositions of bismuth, the surface tension decreases with the increase of temperature, whereas bismuth addition in amount 0.1–0.2 mole an opposite tendency is observed $(\frac{d\sigma }{dT}) > 0$, especially for section *x*_Sn_:*x*_Ag_:*x*_Cu_ = 1:1:2. The same results were obtained with Muggianu and Hillert models.

The surface tension of Sn-Ag-Cu-Bi quaternary alloys decreases as a function of Bismuth in the composition range 0.1 ≤ *x*_Bi_ ≤ 0.7. While with a Bi addition in the range of 80 to 90 atomic percent an opposite trend has been observed. It should be noted that a similar behavior has been previously reported by Kucharski and Fima^22^ for the binary Ag-Bi. The surface tension decreases with the increase of Bi in the binary Ag-Bi. The pure surface tension Bi lower than that of the pure Sn means that increasing the content of Bi in the Sn-Bi welds decreases their surface tension. This effect can be explained by the enrichment of Bi in the superficial layer. Since bismuth has the lowest surface tension of the four alloy metals (σBi < σSn < σAg < σCu), the system seems to reduce its energy by separating the component with the small surface tension at the surface^36^. Indeed, the bismuth component plays an important role not only in reducing the melting temperature of the alloy but also in reducing the surface tension of the SnAgCuBi alloys. It is important to note that the same behavior was observed for the other models (Muggianu and Hillert) and the Guggenheim equation.

**Figure 4:**
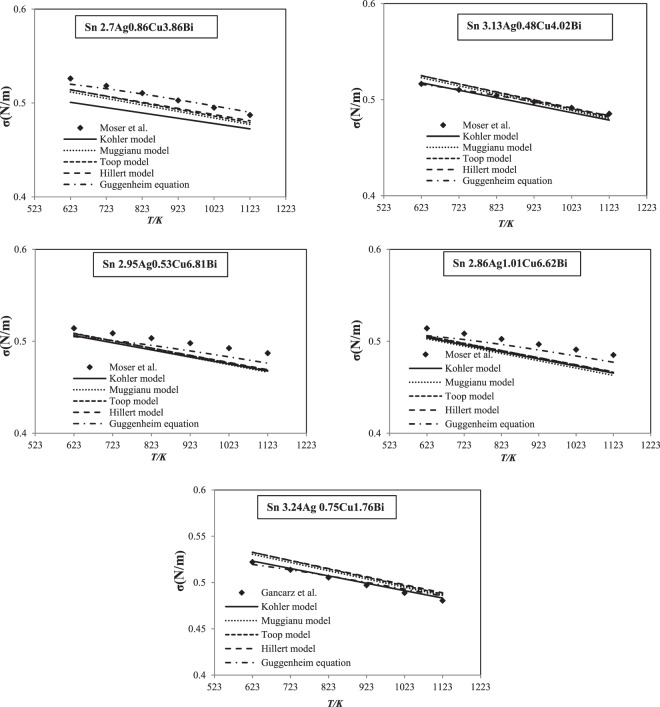
Comparison of experimental surface tensions (Moser *et al*.^11^ and Gancarz *et al*.^14^) of the Sn-Ag-Cu-Bi quaternary system with the calculated ones (this work).

Our calculated surface tensions of five quaternary Sn-Ag-Cu-Bi alloys are illustrated in Fig. 4 together with the measured ones^11,14^ at 623 K–1123 K. It can be seen that a good agreement between the theoretical and measured values is obtained. In addition, for the comparison check, we calculated the mean square deviation corresponding to the experimental results for each traditional model and the Guggenheim equation.

20 $$S=\frac{1}{N}\sqrt{\mathop{\sum }\limits_{i=1}^{N}{({\sigma }_{th,i}-{\sigma }_{\exp ,i})}^{2}}$$

where *σ*_*th,i*_ and *σ*_*exp,i*_ represent the surface tension of Sn-Ag-Cu-Bi alloys a permanent composition i for a theoretical models and an experimental values, respectively, while N is the total amount of investigate alloys. The calculations of square deviation are collected in Table 5.

**Table 5:** Calculation square deviation values S between theoretical and experimental^11,14^ surface tension (N/m).

*T/K*	S_Guggenheim_	S_Kohler_	S_Muggianu_	S_Toop_	S_Hillert_
**Moser** ***et al*** *.* ^11^					
623	0.0034	0.0071	0.0052	0.0045	0.0046
723	0.0026	0.0070	0.0087	0.0046	0.0046
823	0.0024	0.0071	0.0060	0.0049	0.0049
923	0.0026	0.0072	0.0066	0.0055	0.0053
1023	0.0029	0.0076	0.0073	0.0062	0.0059
1123	0.0034	0.0080	0.0080	0.0070	0.0066
**Gancarz** ***et al*** *.* ^14^					
623	0.0024	0.0010	0.0084	0.0106	0.0104
723	7.71*10^−6^	0.0070	0.0118	0.0133	0.0118
823	0.0017	0.0023	0.0075	0.0096	0.0095
923	0.0031	0.0029	0.0072	0.0093	0.0092
1023	0.0042	0.0053	0.0078	0.0095	0.0094
1123	0.0051	0.0060	0.0076	0.0092	0.0091

Generally, the surface tensions calculated with Guggenheim equations are in good accord with those obtained experimentally^11,14^. The calculated values from geometric models are slighly superior than the experimental ones. With the exception of certain temperatures, geometric models are in good accord with experimental data.

### Molar volume in the liquid Sn-Ag-Cu-Bi quaternary alloys

The molar volume of Sn-Ag-Cu-Bi in the liquid phase over a wide temperature range (from 923 to 1423 K) was calculated using geometric models (Kohler, Muggianu, Toop and Hillert).

**Figure 5:**
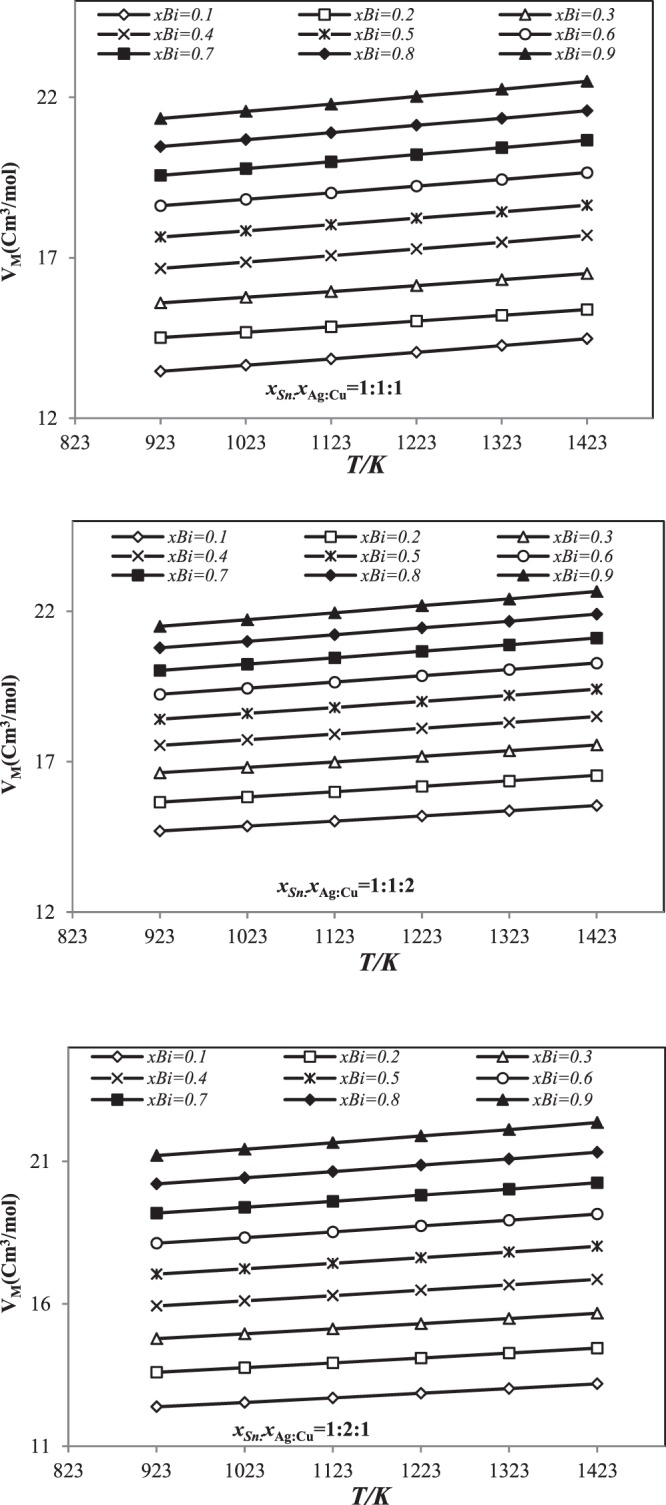
Linear dependences of Molar volume for three sections (*x*_Sn_:*x*_Ag_:*x*_Cu_ = 1:1:1, 1:1:2 and 1:2:1 using Kohler’s model in temperature range 923 *K*–1423 *K*.

We give only the calculated results of the molar volume using the Kohler model (Fig. 5).

It can be noted in Fig. 5 that the molar volume of Sn-Ag-Cu-Bi augments linearly with the rise in temperature. Besides, the rise in the quantity of bismuth has an effect on the molar volume of Sn-Ag-Cu-Bi quaternary system. The similar behavior was observed for other models (Muggianu, Toop and Hillert).

### Density in the liquid Sn-Ag-Cu-Bi quaternary alloys

The density of liquid Sn-Ag-Cu-Bi alloys as a function of temperature along the three sections *x*_Sn_:*x*_Ag_:*x*_Cu_ = 1:1:1, 1:1:2 and 1:2:1 was calculated from the molar volume.

**Figure 6:**
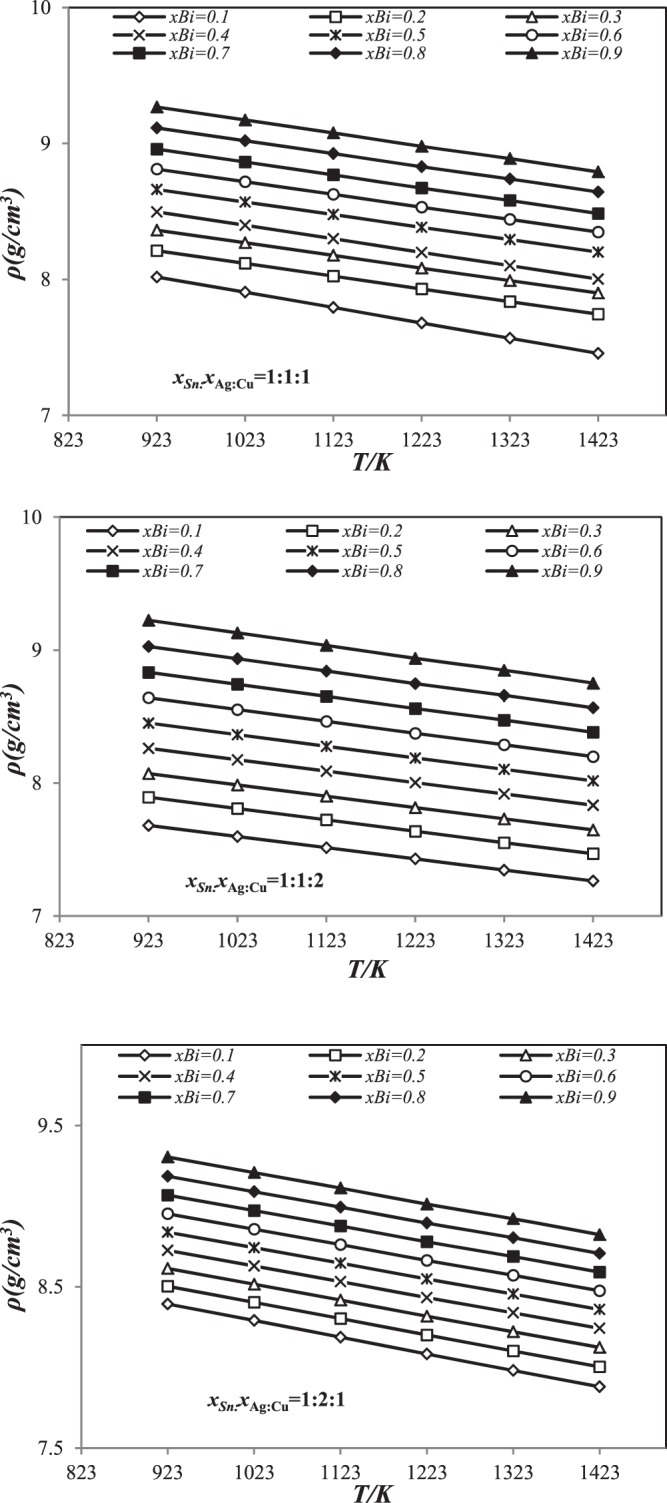
Linear dependences of density for three sections (*x*_Sn_:*x*_Ag_:*x*_Cu_=1:1:1, 1:1:2 and 1:2:1 using Kohler’s model in temperature range 923 *K*–1423 *K*.

As an example, we present only the results of Kohler. The values are illustrated in the Fig. 6. The results obtained clearly show that the density of the quaternary Sn-Ag-Cu-Bi system decreases linearly with temperature $(\frac{d\rho }{dT}) < 0$ and increases with the concentration of Bismuth at a given temperature (Fig. 6). The increase in density with addition of Bi was observed. The same effect has been observed by other authors^11^. This effect can be interpreted as bismuth having the most robust density as tin (ρSn <ρBi).

**Figure 7:**
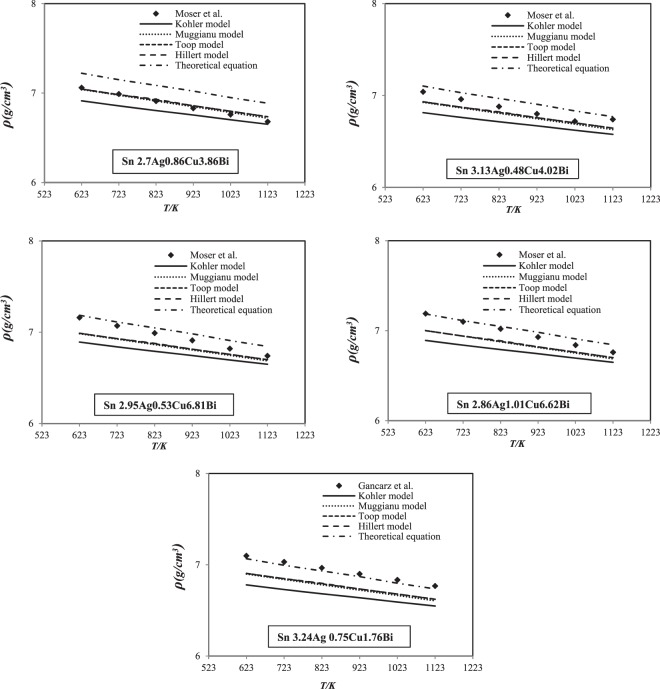
Comparison of experimental density of the Sn-Ag-Cu-Bi quaternary alloys^11,14^ with those calculated values at different temperatures.

The calculated results are compared with those of Moser *et al*.^11^ and Gancarz *et al*.^14^ for (Sn 2.7 Ag 0.86 Cu 3.86 Bi, Sn 3.13 Ag 0.48 Cu 4.02 Bi, Sn 2.95 Ag 0.53 Cu 6.81 Bi and Sn 2.68 Ag 1.01 Cu 6.62 Bi) and (Sn 3.24 Ag 0.75 Cu 1.76 Bi) quaternary alloys^11,14^, respectively (Fig. 7).

It can be noted simply from Fig. 7, our calculated density results in this study using the theoretical equation is in good accord with the experimental data. However, the geometric models are higher than experimental one.

It may be concluded that the estimated density values for the quaternary Sn-Ag-Cu-Bi using theoretical equation are characteristics to ideal solution at different temperatures (623 K–1123 K).

Standard deviations were determined for all models and for theoretical equation as:

21 $$S=\frac{1}{N}\sqrt{\mathop{\sum }\limits_{i=1}^{N}{({{\rm{\rho }}}_{th,i}-{{\rm{\rho }}}_{\exp ,i})}^{2}}$$

where ρ_*th*,*i*_ and ρ_exp,*i*_ represent the density of Sn-Ag-Cu-Bi alloys a permanent composition i for a theoretical models and an experimental values, respectively, while N is the totality amount of investigate alloys. The calculations of square deviation are collected Table 6.

**Table 6:** Calculation square deviation values S between theoretical and experimental^11,14^ density (g/cm^3^).

*T/K*	S_Theoretical equation_	S_Kohler_	S_Muggianu_	S_Toop_	S_Hillert_
**Moser** ***et al*** *.* ^14^					
623	0.0536	0.1087	0.0584	0.0574	0.0573
723	0.0450	0.1053	0.0593	0.0573	0.0573
823	0.0518	0.0903	0.0513	0.0484	0.0460
923	0.0591	0.0725	0.0418	0.0385	0.0385
1023	0.0620	0.0555	0.0306	0.0270	0.0270
1123	0.0620	0.0546	0.0373	0.0328	0.0328
**Gancarz** ***et al*** *.* ^14^					
623	0.0315	0.3180	0.2002	0.1926	0.1926
723	0.0371	0.3032	0.1936	0.1845	0.1846
823	0.0334	0.2837	0.1861	0.1747	0.1695
923	0.0300	0.2614	0.1783	0.1655	0.1695
1023	0.0357	0.2422	0.1703	0.1550	0.1550
1123	0.0320	0.2201	0.1631	0.1457	0.1457

It can be seen from S that the calculated values of the density using the (Toop and Hillert) models give results close to experimental values^11^, in particular for temperature range at 823–1123 K. While for temperature range 623 K–1123 K, the results calculated using the theoretical equation give the values closest to the experiment data^14^.

### Viscosity in the liquid Sn-Ag-Cu-Bi quaternary alloys

**Figure 8:**
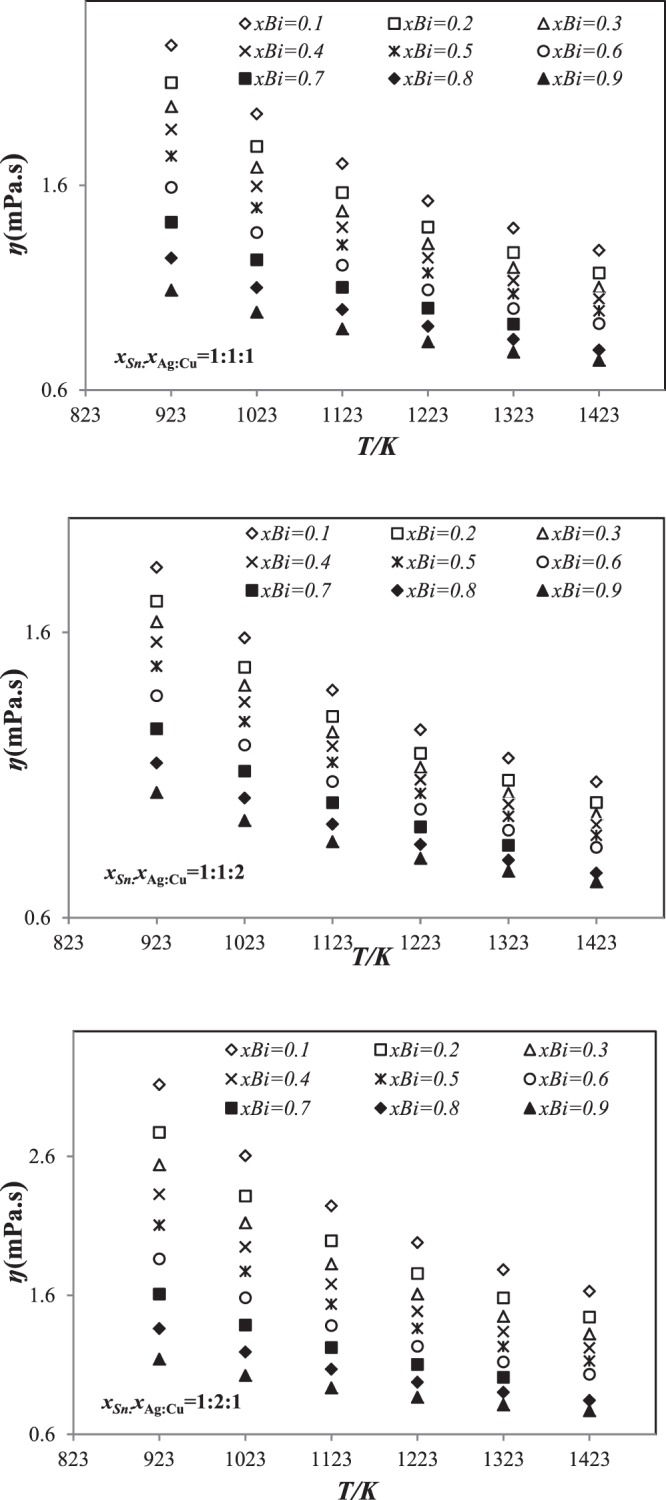
Temperature dependence of the viscosity (mPa/s) for three sections (*x*_Sn_:*x*_Ag_:*x*_Cu_=1:1:1, 1:1:2 and 1:2:1 using Seetharaman-Sichen equation in the temperature range 923 *K*–1423 *K*.

**Figure 9:**
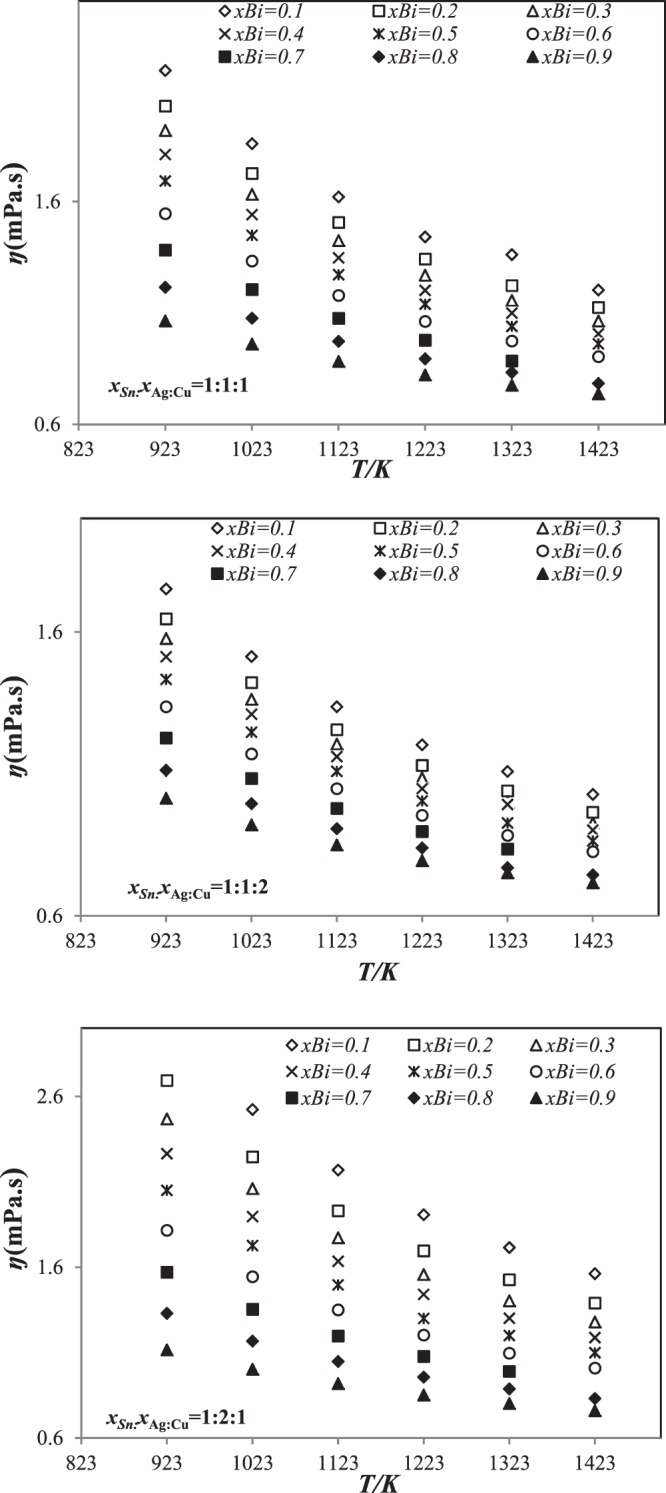
Temperature dependence of the viscosity (mPa/s) for three sections (*x*_Sn_:*x*_Ag_:*x*_Cu_=1:1:1, 1:1:2 and 1:2:1 using Kaptay equation in the temperature range 923 *K*–1423 *K*.

Based on Seetharaman-Sichen statistic and Kaptay equations for Sn-Ag-Cu-Bi alloys, viscosities were calculated. The theoretical results for the viscosity of Sn-Ag-Cu-Bi alloys obtained using Seetharaman-Sichen and Kaptay equations are presented in Tables 7 and 8, respectively as an Arrhenius equation at various temperatures and given away in Figs 8 and 9.

**Table 7:** Temperature dependence of the viscosity (mPa/s) of Sn-Ag-Cu-Bi quaternary alloys at various temperatures using Seetharaman-Sichen equation.

Alloy (at.%)				${\boldsymbol{\eta }}=\exp (\frac{{\boldsymbol{E}}}{{\boldsymbol{RT}}})$	
*x*_Sn_	*x*_Ag_	*x*_Cu_	*x*_Bi_	A	E
***x***_**Sn**_ **:** ***x***_**Ag**_ **:** ***x***_**Cu**_ **=1:1:1**					
0.3334	0.3333	0.3333	0.0000	0.5565	12437.74
0.3000	0.3000	0.3000	0.1000	0.4421	12595.71
0.2666	0.2667	0.2667	0.2000	0.3981	12770.30
0.2334	0.2333	0.2333	0.3000	0.3723	12845.13
0.2000	0.2000	0.2000	0.4000	0.3559	12745.36
0.1666	0.1667	0.1667	0.5000	0.3457	12421.11
0.1334	0.1333	0.1333	0.6000	0.3406	11830.82
0.1000	0.1000	0.1000	0.7000	0.3409	10949.53
0.0666	0.0667	0.0667	0.8000	0.3485	9777.260
0.0334	0.0333	0.0333	0.9000	0.3704	8275.750
0.0000	0.0000	0.0000	1.0000	0.4453	6450.001
***x***_**Sn**_ **:** ***x***_**Ag**_ **:** ***x***_**Cu**_ **=1:1:2**					
0.5000	0.2500	0.2500	0.0000	0.4906	11539.83
0.4500	0.2250	0.2250	0.1000	0.4045	11581.40
0.4000	0.2000	0.2000	0.2000	0.3749	11647.91
0.3500	0.1750	0.1750	0.3000	0.3598	11631.28
0.3000	0.1500	0.1500	0.4000	0.3509	11481.63
0.2500	0.1250	0.1250	0.5000	0.3461	11165.70
0.2000	0.1000	0.1000	0.6000	0.3443	10650.23
0.1500	0.0750	0.0750	0.7000	0.3464	9926.916
0.1000	0.0500	0.0500	0.8000	0.3541	8987.113
0.0500	0.0250	0.0250	0.9000	0.3741	7835.113
0.0000	0.0000	0.0000	1.0000	0.4453	6450.001
***x***_**Sn**_ **:** ***x***_**Ag**_ **:** ***x***_**Cu**_ **=1:2:1**					
0.2000	0.4000	0.4000	0.0000	0.6363	14083.91
0.1800	0.3600	0.3600	0.1000	0.4921	14167.05
0.1600	0.3200	0.3200	0.2000	0.4325	14258.51
0.1400	0.2800	0.2800	0.3000	0.3965	14250.19
0.1200	0.2400	0.2400	0.4000	0.3726	14067.28
0.1000	0.2000	0.2000	0.5000	0.3566	13626.64
0.0800	0.1600	0.1600	0.6000	0.3471	12903.32
0.0600	0.1200	0.1200	0.7000	0.3440	11839.13
0.0400	0.0800	0.0800	0.8000	0.3495	10425.75
0.0200	0.0400	0.0400	0.9000	0.3704	8538.246
0.0000	0.0000	0.0000	1.0000	0.4453	6450.001
**(Sn-Ag-Cu)+Bi**					
0.9678	0.0276	0.0046	0.0000	0.4151	7018.678
0.9237	0.0313	0.0048	0.0402	0.3894	7145.051
0.8971	0.0295	0.0053	0.0681	0.3863	7130.086
0.9613	0.0313	0.0074	0.0000	0.4118	7176.644
0.8808	0.0720	0.0086	0.0386	0.3753	8141.900
0.8951	0.0286	0.0101	0.0662	0.3848	7212.395
0.9425	0.0324	0.0075	0.0176	0.4244	7216.552
0.9330	0.0310	0.0005	0.0310	0.3921	7135.074

**Table 8:** Temperature dependence of the viscosity (mPa/s) of Sn-Ag-Cu-Bi quaternary alloys at various temperatures using Kaptay equation.

Alloy (at.%)				${\boldsymbol{\eta }}=\exp (\frac{{\boldsymbol{E}}}{{\boldsymbol{RT}}})$	
*x*_Sn_	*x*_Ag_	*x*_Cu_	*x*_Bi_	A	E
***x***_**Sn**_ **:** ***x***_**Ag**_ **:** ***x***_**Cu**_ **=1:1:1**					
0.3334	0.3333	0.3333	0.0000	0.6071	10849.77
0.3000	0.3000	0.3000	0.1000	0.4164	12720.42
0.2666	0.2667	0.2667	0.2000	0.3775	12911.64
0.2334	0.2333	0.2333	0.3000	0.3570	12911.64
0.2000	0.2000	0.2000	0.4000	0.3419	12803.56
0.1666	0.1667	0.1667	0.5000	0.3375	12379.54
0.1334	0.1333	0.1333	0.6000	0.3352	12379.54
0.1000	0.1000	0.1000	0.7000	0.3292	11323.66
0.0666	0.0667	0.0667	0.8000	0.3478	9594.350
0.0334	0.0333	0.0333	0.9000	0.3724	8059.591
0.0000	0.0000	0.0000	1.0000	0.4448	6449.169
***x***_**Sn**_ **:** ***x***_**Ag**_ **:** ***x***_**Cu**_ **=1:1:2**					
0.5000	0.2500	0.2500	0.0000	0.4373	12180.01
0.4500	0.2250	0.2250	0.1000	0.3832	11664.54
0.4000	0.2000	0.2000	0.2000	0.3587	11697.79
0.3500	0.1750	0.1750	0.3000	0.3468	11622.97
0.3000	0.1500	0.1500	0.4000	0.3559	11082.56
0.2500	0.1250	0.1250	0.5000	0.3385	11074.24
0.2000	0.1000	0.1000	0.6000	0.3392	10525.52
0.1500	0.0750	0.0750	0.7000	0.3433	9768.950
0.1000	0.0500	0.0500	0.8000	0.3443	9012.376
0.0500	0.0250	0.0250	0.9000	0.3760	7613.129
0.0000	0.0000	0.0000	1.0000	0.4448	6449.169
***x***_**Sn**_ **:** ***x***_**Ag**_ **:** ***x***_**Cu**_ **=1:2:1**					
0.2000	0.4000	0.4000	0.0000	0.5798	14624.32
0.1800	0.3600	0.3600	0.1000	0.4611	14449.73
0.1600	0.3200	0.3200	0.2000	0.4098	14449.73
0.1400	0.2800	0.2800	0.3000	0.3798	14366.59
0.1200	0.2400	0.2400	0.4000	0.3602	14117.17
0.1000	0.2000	0.2000	0.5000	0.3436	13701.47
0.0800	0.1600	0.1600	0.6000	0.3412	12828.50
0.0600	0.1200	0.1200	0.7000	0.3409	11722.74
0.0400	0.0800	0.0800	0.8000	0.3488	10259.47
0.0200	0.0400	0.0400	0.9000	0.3723	8422.082
0.0000	0.0000	0.0000	1.0000	0.4444	6449.169
**(Sn-Ag-Cu)+Bi**					
0.9678	0.0276	0.0046	0.0000	0.4334	6523.164
0.9237	0.0313	0.0048	0.0402	0.4013	6607.135
0.8971	0.0295	0.0053	0.0681	0.3981	6590.507
0.9613	0.0313	0.0074	0.0000	0.4325	6622.932
0.8808	0.0720	0.0086	0.0386	0.3941	7383.663
0.8951	0.0286	0.0101	0.0662	0.3981	6627.089
0.9425	0.0324	0.0075	0.0176	0.4045	6555.589
0.9330	0.0310	0.0005	0.0310	0.4037	6600.484

As seen from Fig. 8, the viscosity of these alloys decreases curvilinearly with increasing temperature and bismuth. This decrease of viscosity by adding bismuth can be explained by its low viscosity in comparison of the other metals (ŋBi < ŋSn < ŋAg < ŋCu).

**Figure 10:**
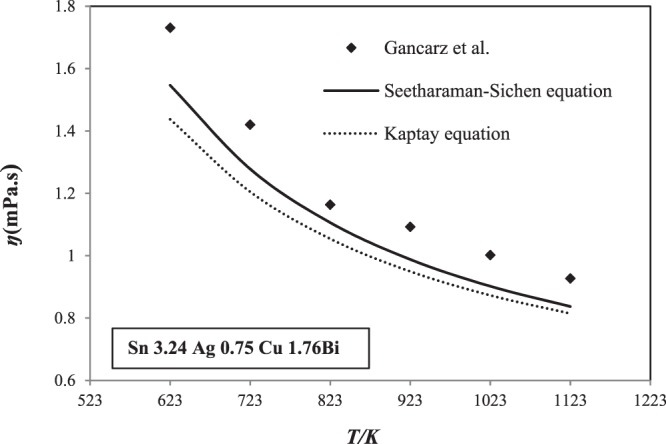
Comparison of the calculated viscosity of Sn-Ag-Cu-Bi alloys using Seetharaman-Sichen and Kpatay equations with those experimental values^14^ at different temperatures.

The results show a similar behavior of the viscosity in function of the temperature than those obtained using Seetharaman-Sichen equation. The viscosity decreases curvilinear with increasing temperature, and decreases with function of temperature and Bi content for all sections (Fig. 9). The predicted results are compared to each other and to the experimental ones (Fig. 10).

As seen in Fig. 10, the viscosity values obtained by Seetharamn sichen and Kaptay equations are slightly lower than those measured by Gancarz *et al*.^14^ for Sn 3.24 Ag 0.75 Cu 1.76 Bi alloys.

## Conclusions

In this work the some thermo-physical properties (Surface tension, molar volume, density and viscosity) of the quaternary system Sn-Ag-Cu-Bi have been predicted at different temperatures. Therefore, we reformulated models, which permit one to calculate the surface tension, molar volume and density. This study is carried out using traditional geometric models and theoretical equation, such as Kohler, Muggianu, Toop and Hillert as symmetric and asymmetric models and Guggenheim, Seetharaman-sichen and Kaptay equations. Some important results of our predictions reveal the following conclusions:

- For all compositions of Bismuth (except for the composition range with Bismuth molar content lower than *x*_Bi_ = 0.2) in the quaternary system Sn-Ag-Cu-Bi, the surface tension decreases with increasing temperature. Indeed, the surface tension diminishes with addition of Bismuth concentration.
- Addition of Bismuth to ternary Sn-Ag-Cu increases the molar volume and density but diminishes the viscosity.
- We can conclude that among all the traditional predictive models and the theoretical equation, Guggnheim (for surface tension) and the theoretical equation (for viscosity) give the best agreement with the experimental one.
- The viscosity results of the present work obtained by Seetharamn sichen and Kaptay equations are slightly lower than those measured by Gancarz *et al*.^14^.
